# Equity assessment of the distribution of mental health beds in China: based on longitudinal data from 2011 to 2020

**DOI:** 10.1186/s12913-022-08658-z

**Published:** 2022-11-30

**Authors:** Xin Fan, Weibo Zhang, Yanping Guo, Jun Cai, Bin Xie

**Affiliations:** 1 Shanghai Mental Health Center, Shanghai Jiao Tong University School of Medicine, 200030 Shanghai, China; 2 Center for Mental Health Management, China Hospital Development Institute, Shanghai Jiao Tong University, 200030 Shanghai, China; 3 Shanghai Center for Mental Disease Control and Prevention, 200030 Shanghai, China

**Keywords:** Mental health bed, Resource allocation, Equity, Gini coefficient, Lorenz curve, Theil index

## Abstract

**Background:**

Mental health problems have become a public health problem that needs to be solved in China. However, medical resources for mental healthcare remain insufficient and unevenly distributed. The Chinese central government has taken many measures to address this issue over the last decade. This study aimed to evaluate the changes in equity in mental health bed allocation from 2011 to 2020.

**Methods:**

The data of this study came from the China Health Statistical Yearbook (2012–2021) and the China National Administrative Division Information Platform. The annual growth rate was used to evaluate the time trends of mental health beds. The Lorenz curve, Gini coefficient and Theil index were used to assess equity in the demographic and geographical dimensions. The distribution of mental health beds was visualized on a map using geographic information system (GIS) software.

**Results:**

The total number of mental health beds in China increased steadily from 2011 to 2020. At the national level, the Gini coefficient and Theil index all exhibited downward trends over time. The mental health bed allocation in terms of the demographic dimension was relatively equitable, with Gini values all less than 0.3; however, the Gini coefficients by geographical area were all more than 0.6, indicating inequity. Intraregional contribution rates were higher than interregional contribution rates, which were all above 60%.

**Conclusion:**

The overall distribution equity of mental health beds improved from 2011 to 2020. The equity of mental health beds in terms of population size is superior to that in terms of geographical area. Intraregional differences are the main source of inequity. In particular, differences within the western region need to be given attention. Thus, the findings from this study emphasize that the demographic and geographical distributions and all influencing factors should be considered when the government makes mental health resource allocation policies.

## Background

Mental health, ignored for far too long, is crucial to the overall well-being of individuals, societies, and countries. In recent years, mental health issues have become more prevalent, impacting people of all ages and financial levels worldwide[1]. Globally, three-quarters of the mental, neurological, and substance use disorder burden was experienced by people from developing countries; China alone accounted for nearly 17% of the global burden[2]. Furthermore, according to a survey conducted in 31 Chinese provinces, the lifetime prevalence of mental disorders among adults was 16.6%[3]; given China’s population of 1.4 billion, this prevalence ratio suggests that many people are affected by mental health problems.

The reduction in the mental disorder burden in China is essential and urgent. However, the resources provided to tackle the enormous burden, such as financial investment[4, 5], professional staff[6, 7], and medical institutions[8–10], remain insufficient and unevenly distributed. According to a National Health Commission report, two-thirds of China’s counties and districts do not have mental health institutions. Moreover, mental health resources are mainly located in the eastern coastal provinces[5]. A Chinese Center for Mental Disease Control and Prevention survey found that 47.21% of mental health institutions, 42.06% of beds, and 46.22% of professional staff are concentrated in East China. Surprisingly, in ten provinces (Shanxi, Inner Mongolia, Jilin, Heilongjiang, Anhui, Tibet, Gansu, Qinghai, Ningxia, and Xinjiang), more than 50% of counties have no mental health institutions[8].

The Chinese government has prioritized ensuring that all citizens’ right to health is protected. Over the past ten years, it has implemented corresponding measures to enrich mental health resources and address health resource maldistribution[11]. First, making a better mental health system has been incorporated into the national health system reform. Much policy and financial support has been given to build mental health institutions and attract professionals, especially in West and Central China[12]. Second, ten national ministries and commissions, such as the Ministry of Health, the National Commission of Development and Reform, the Ministry of Finance, and the Ministry of Civil Affairs, have jointly formulated the National Mental Health Work Plan[10]. The plan specifies targets for resource allocation in different regions and strategies to ensure implementation. Finally, the National Mental Health Law of the People’s Republic of China was adopted by the National People’s Congress on October 26, 2012, and it took effect on May 1, 2013. Different sections of the law discuss the prevention and rehabilitation of mental disorders, the financing and management of services, the provision of social welfare services for patients and their families, and the responsibilities of different agencies and community members in the mental health effort[13]. Many scholars agree that the law will play an unparalleled role in establishing a better mental health service system in China[13, 14].

Mental health beds are a pivotal component of mental healthcare resources. How to optimize the allocation of mental health beds has been a concern of policymakers and scholars in different countries[15–17], including China. Moreover, as a rule in China, the hospital bed is the anchor for the deployment of health professionals. For example, according to the Basic Standards for Medical Institutions issued by the Ministry of Health, a mental hospital needs to meet the standard of 0.4–0.55 professionals per bed[18]. Understanding the current state of mental health bed allocation, particularly the equity of allocation, is an issue that cannot be ignored in the development and optimization of health policy. In recent years, researchers have begun to focus on this issue. Many studies have investigated mental health bed allocation quantity and inequity in China, and they have observed disparities in mental health beds across different regions[4, 6, 8, 16, 19]. However, they did not evaluate trends in changes in mental health bed allocation equity over time and its relation to China’s mental health laws and policies in the last ten years. Existing research primarily focuses on measuring the degree of inequity in the demographic distribution. We believe geographical area should be considered, as geographical accessibility is an important aspect of health equity[20, 21]. In terms of research methods, many quantitative indicators are extensively used to measure health resource equity[22–24], including the Gini coefficient, Lorenz curve, Theil index, Concentration index, and Atkinson index. These indicators are straightforward and practical. For example, when paired with Gini coefficients, the Lorenz curve might vividly show equality in resource allocation[25]; the Theil index could reflect the contribution rate within and between groups when examining the major reasons for inequity[26]. Nevertheless, mental health policy researchers have paid little attention to these methods.

Therefore, in cases of nonuniform distributions of mental health care needs across regions, we aimed to evaluate the trend of equity in mental health bed allocation in not only demographic but also geographic terms in China over the last ten years, from 2011 to 2020. The Lorenz curve, Gini coefficient, and Theil index were used in this study. The findings of this study may help reflect the impact of the government’s efforts to improve health resources and provide a theoretical foundation for policymakers to take appropriate measures to optimize the allocation of mental health beds.

## Methods

### Data collection

We collected data from 2011 to 2020 to track the changes in the allocation of mental health beds in China. All data referred to are available in the public domain. The mental health beds and demographic data were extracted from the China Health Statistics Yearbooks 2012 to 2021[27], while the geographic area data were gathered from the China National Administrative Division Information Platform[28].

**Fig. 1:**
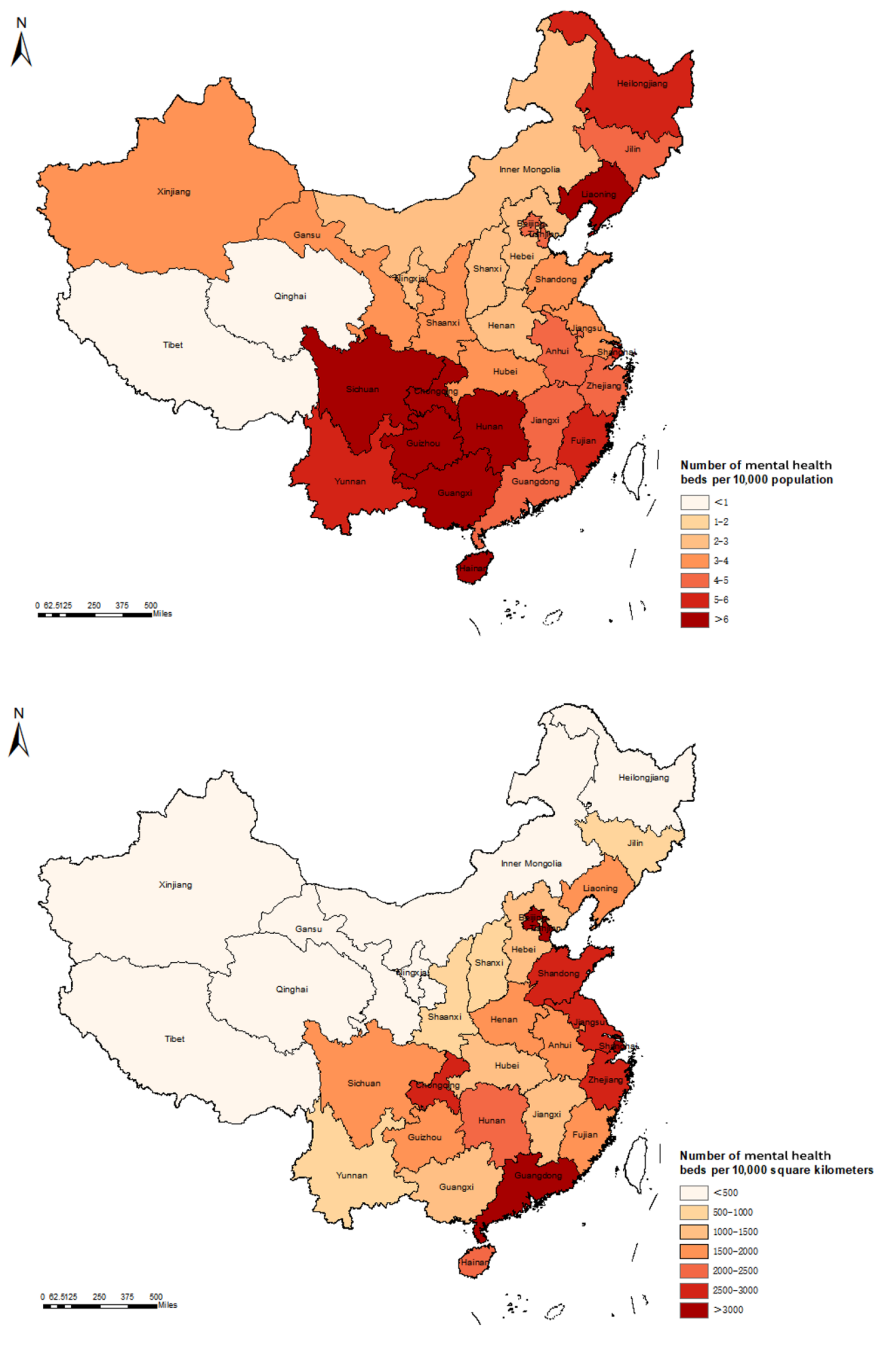
Demographic and geographic distribution of mental health beds at the provincial level in China in 2020. **A** shows the distribution of the number of mental health beds per 10,000 population, and Sichuan had the largest number of mental health beds per 10,000 population. **B** shows the distribution of the number of mental health beds per 10,000 population, and Shanghai had the largest number of mental health beds per 10,000 square kilometres

China has 34 provincial-level administrative regions (hereafter collectively referred to as provinces), as shown in Fig. 1. We analysed the data of the 31 provinces in mainland China and excluded the Hong Kong and Macao special administrative regions and Taiwan province due to the inconsistent quality of the statistics. According to the economic development level and geographical position, these 31 provinces were divided into three groups: eastern, central and western regions. The eastern region included Beijing, Tianjin, Hebei, Liaoning, Shanghai, Jiangsu, Zhejiang, Fujian, Shandong, Guangdong and Hainan (11 provinces). The central region included Shanxi, Jilin, Heilongjiang, Anhui, Jiangxi, Henan, Hubei and Hunan (8 provinces). The western region included Inner Mongolia, Chongqing, Guangxi, Sichuan, Guizhou, Yunnan, Tibet, Shaanxi, Gansu, Qinghai, Ningxia and Xinjiang (12 provinces).

### Data analysis

#### Numbers and annual growth rates of mental health beds

For a descriptive analysis of the demographic and geographic distribution of mental health beds, we calculated the densities of mental health beds per 10,000 population and per 10,000 square kilometres at the provincial and regional levels. The annual growth rate (AGR) of mental health beds from 2011 to 2020 was computed as well. The formula of the AGR is as follows:

1 $$AGR=\sqrt[m]{\frac{B}{A}}-1$$

where B represents the quantity of mental health beds in 2020, A represents the quantity of mental health beds in 2011, and m represents the number of years.

### Equity evaluation of the distribution of mental health beds

#### Gini coefficient and Lorenz curve

The Gini coefficient has been used in a wide variety of resource allocation contexts to measure inequity, including income, wealth, credit availability and energy[23]. It is also regarded as a superior tool for evaluating the equity of health resource allocation in the demographic and geographical dimensions[22]. It is derived from the Lorenz curve and indicates the ratio of the area between the Lorenz curve and the 45° line to the whole area below the 45° line. With regard to the Lorenz curve, the x-axis represents the cumulative percentage of population or geography, the y-axis shows the cumulative percentage of the mental health beds, and the 45° line means absolute equity. The larger the distance from the absolute equality curve is, the greater the inequity.

The Gini coefficient is an absolute indicator with a universal grading scale. It takes a value from 0 to 1, with a higher value indicating greater inequity[29]. Generally, a Gini coefficient that is smaller than 0.2 indicates a very low inequity level, while a Gini coefficient larger than 0.4 indicates high inequity[30]. The formula to calculate the Gini coefficient is presented as follows:

2 $$G=1-{\sum }_{i=0}^{n-1}\left({X}_{i+1}-{X}_{i}\right)\left({Y}_{i+1}+{Y}_{i}\right)$$

In formula 2, *G* represents the value of the Gini coefficient; *Xi* is the cumulative percentage of population or geographic area in the *i*th province; *Yi* is the cumulative percentage of mental health beds in the *i*th province; and *n* is the total number of provinces.

### Theil index

The Theil index is another common measure of inequity that has some advantages over the Gini coefficient. The Gini coefficient can only describe the degree of equity, while the Theil index can be used to analyse the source of inequity[31]. However, the Theil index is a relative indicator with no universal assessment standard. The scope of this index is from 0 to 1. The smaller the value is, the more equity in allocation[32]. We apply the Theil index to confirm intraregional and interregional inequity between and within the eastern, central, and western regions in China. The Theil index is calculated as follows:

3 $$T={\sum }_{i=1}^{n}{P}_{i}log\frac{{P}_{i}}{{Y}_{i}}$$

In formula 3, *P*_*i*_ represents the proportion of the *i*th province’s population (area) out of the total population (area); *Y*_*i*_ represents the proportion of health resources owned by the *i*th province out of the total number of mental health beds. The total Theil index can be decomposed into the intra-Theil index and inter-Theil index. Formula 4 and formula 5 are used to calculate the intra-Theil index and inter-Theil index, respectively.

4 $${T}_{intra}={\sum }_{g=1}^{k}{P}_{g}{T}_{g}$$

5 $${T}_{inter}={\sum }_{g=1}^{k}{P}_{g}log\frac{{P}_{g}}{{Y}_{g}}$$

6 $$T={T}_{intra}+{T}_{inter}$$

In the above formulas, *T*_*intra*_ means the differences in mental health bed allocation in the region; *T*_*inter*_ means the differences in mental health bed allocation between regions; *Pg* represents the proportion of the *g*th region’s population (area) accounting for the total population (area); and *Yg* represents the proportion of mental health beds owned by the *g*th region accounting for the total number of mental health beds. *k* means the total number of regions. The contribution rates of *T*_*intra*_ and *T*_*inter*_ can be calculated by dividing the total Theil index.

All statistical computations were conducted in Microsoft Excel 2020 (Microsoft Corporation, Redmond, WA, USA), while ArcGIS software (Redlands, California, USA) was used to map the geographical distribution of mental health beds.

## Result

### Current situation of mental health bed allocation in China

Table 1 shows the numbers of mental health beds in the 31 regions of China in 2020 and the annual growth rate of mental health beds per 10,000 population/square kilometres from 2011 to 2020. As of the end of 2020, the total number of mental health beds in China was approximately 652,939, and the average number of mental health beds per 10,000 population and per 10,000 square kilometres in the 31 provinces was 4.62 and 683.34, respectively. The range of mental health beds per 10,000 population was between 0.36 (Tibet) and 9.10 (Sichuan), and the range of mental health beds per 10,000 square kilometres was between 1.10 (Tibet) and 21,830.16 (Shanghai). Figure 1 shows the distribution of mental health beds per 10,000 population and per 10,000 square kilometres at the provincial level in China in 2020.

**Table 1:** Basic distribution of mental health beds in China in 2020 and the annual growth rate of mental health beds from 2011 to 2020

	Total mental health beds(2020)	Mental health beds per 10,000 population (2020)	Annual growth rate of mental health beds per 10,000 population (2011–2020)	Mental health beds per 10,000 km^2^ (2020)	Annual growth rate of mental health beds per 10,000 km^2^ (2011–2020)
Province					
Beijing	8777	4.01	-1.87	5351.83	-0.98
Tianjin	6856	4.94	6.11	5761.34	6.38
Hebei	18,806	2.52	16.38	1004.59	16.77
Liaoning	26,577	6.24	9.19	1794.53	8.84
Shanghai	13,753	5.53	0.07	21830.16	0.71
Jiangsu	26,091	3.08	5.61	2547.95	6.44
Zhejiang	27,945	4.33	8.74	2700.00	10.78
Fujian	22,313	5.37	15.62	1815.54	17.05
Shandong	39,871	3.93	12.27	2520.29	12.92
Guangdong	56,301	4.47	11.51	3231.97	13.78
Hainan	6798	6.74	12.14	2192.90	13.89
Shanxi	9610	2.75	9.16	613.27	8.81
Jilin	12,044	5.00	12.71	633.23	11.06
Heilongjiang	16,519	5.19	12.72	375.69	10.42
Anhui	25,041	4.10	15.96	1793.77	16.25
Jiangxi	21,445	4.75	18.94	1283.36	19.03
Henan	25,322	2.55	12.26	1518.11	12.97
Hubei	23,105	4.00	14.03	1243.54	14.06
Hunan	44,731	6.73	17.00	2111.95	17.10
Inner Mongolia	6292	2.62	11.58	52.60	11.19
Chongqing	21,733	6.78	10.89	2637.50	12.05
Guangxi	31,879	6.36	18.47	1342.84	19.48
Sichuan	76,108	9.10	17.91	1547.85	18.42
Guizhou	29,964	7.77	30.39	1700.57	31.93
Yunnan	23,902	5.06	14.96	606.65	15.21
Tibet	131	0.36	17.74	1.10	20.20
Shaanxi	12,462	3.15	14.60	606.13	15.30
Gansu	7696	3.08	18.17	169.18	17.85
Qinghai	424	0.72	11.00	6.09	11.53
Ningxia	1801	2.50	16.64	271.23	18.20
Xinjiang	8642	3.34	5.72	52.70	7.58
Region					
East	254,088	4.19	8.89	2392.32	9.98
Central	177,817	4.23	14.44	1072.67	14.35
West	221,034	5.77	16.55	323.37	17.27
Total	652,939	4.63	12.57	683.34	13.20

### Trends in the distribution density of mental health beds

As shown in Table 1, the total number of mental health beds in China grew by 13.20% per year (213,877 in 2011 to 652,939 in 2020). The mental health beds in terms of 10,000 population and square kilometres were also increasing. Mental health beds per 10,000 populations nationwide increased from 1.60 to 2011 to 4.63 in 2020. The province with the largest annual growth rate of mental health beds was Guizhou (30.39%, 2474 to 29,964) (Table 1). Beijing and Shanghai showed no growth or even a slowdown in mental health beds over the study period. Mental health beds per 10,000 square kilometres nationwide increased from 223.84 to 2011 to 683.34 in 2020. The province with the largest annual growth rate of mental health beds was Guizhou (31.93%, 140.41 to 1700.51).

**Fig. 2:**
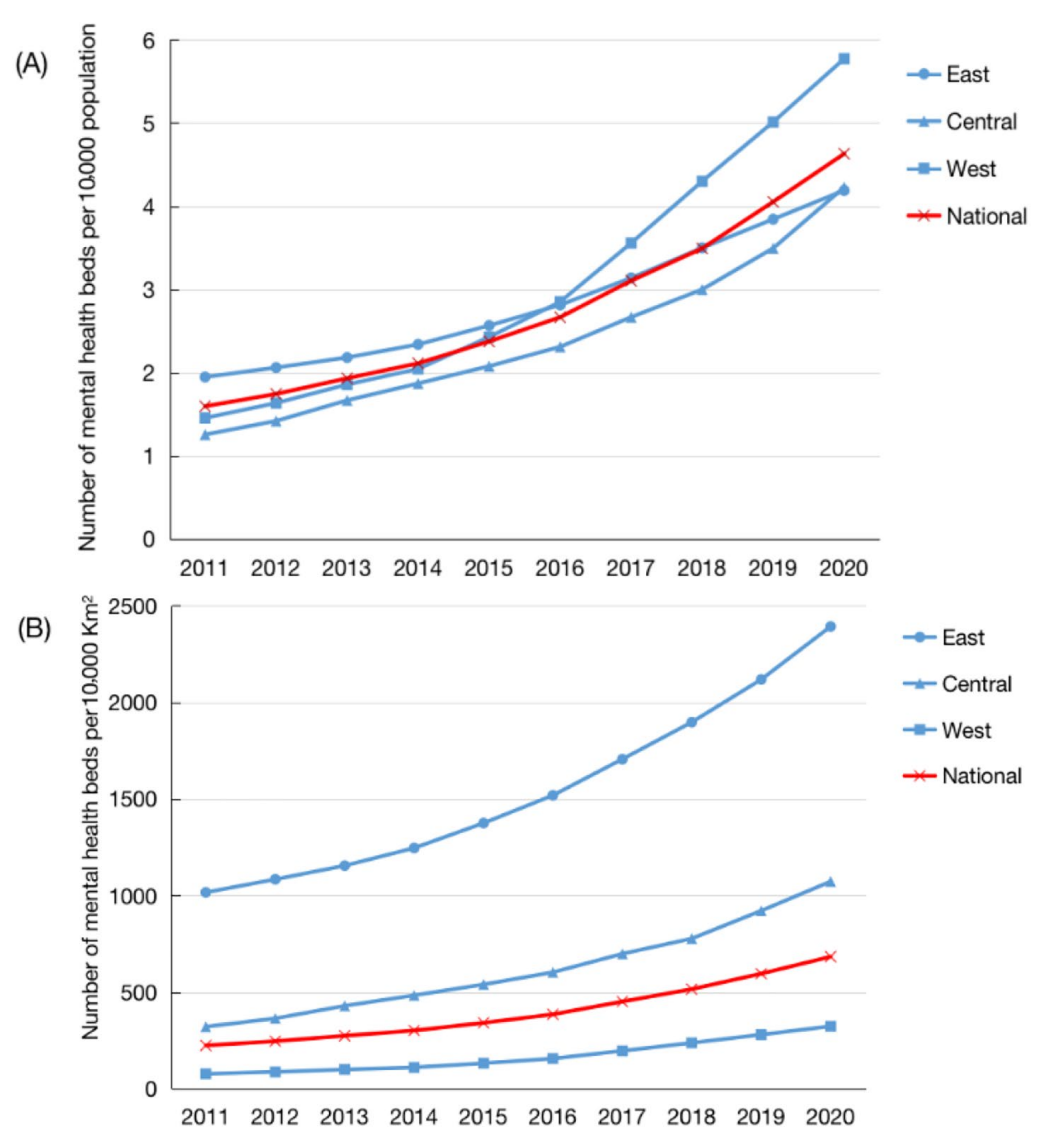
Temporal trends in the numbers of mental health beds in China. **A** shows the mental health beds allocated by population; **B** shows the mental health beds allocated by geographical area

When stratified by region, the largest annual growth rate of mental health beds per 10,000 population and per 10,000 square kilometres was seen in the western region. The highest number of mental health beds per 10,000 people was in the western region. The highest number of mental health beds per 10,000 square kilometres was in the eastern region. The eastern region obtained the highest number of mental health beds per 10,000 people from 2011 to 2015. The western region replaced it as the first from 2016 onwards, with a rapidly increasing number of mental health beds (Fig. 2).

### The equity of mental health bed resource allocation in China

#### Lorenz curves

**Fig. 3:**
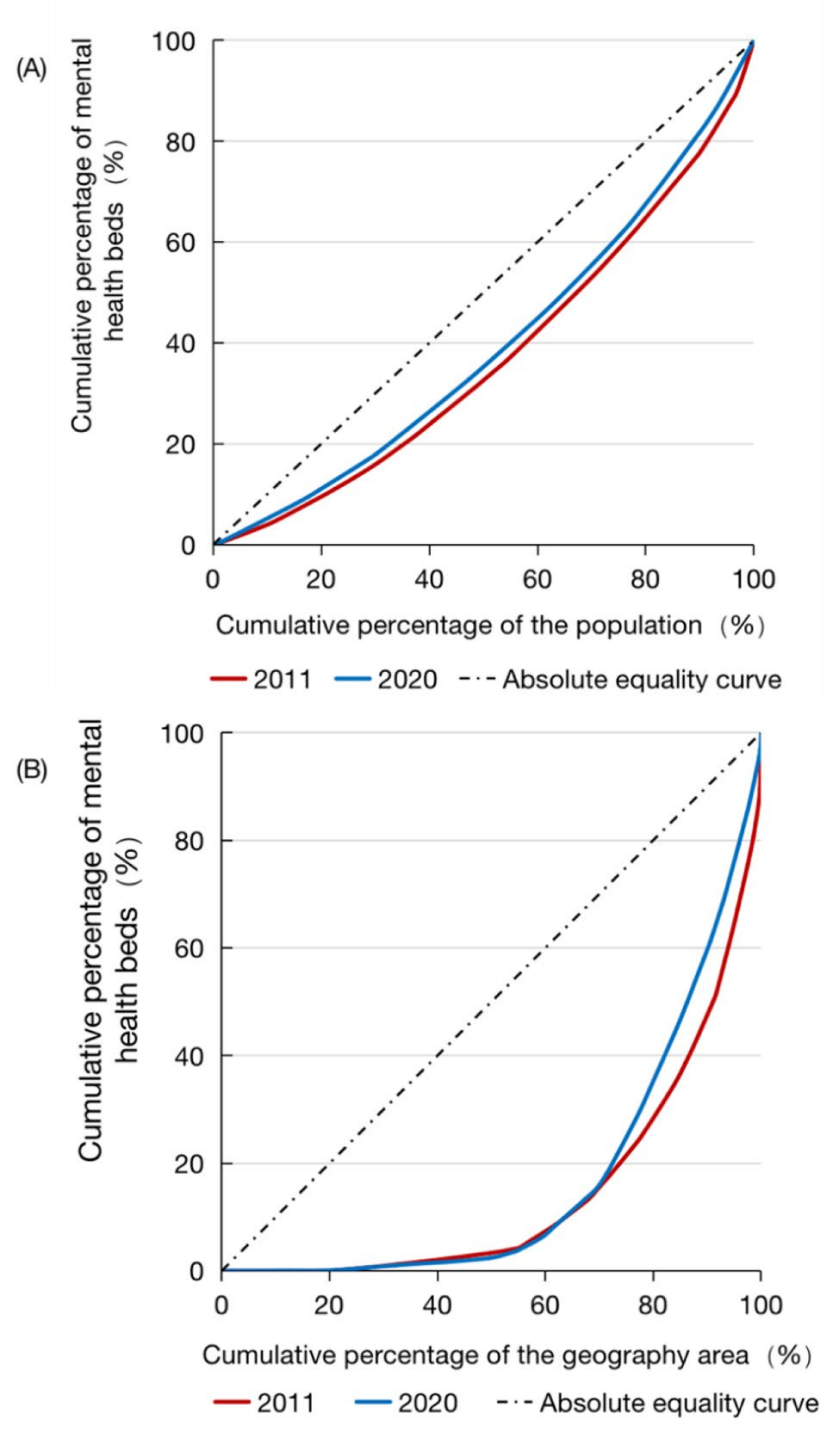
The Lorenz curves of mental health beds in 2011 and 2020. **A** is the demographic dimension; **B** is the geographical dimension

Figure 3 presents the Lorenz curves based on the demographic and geographical dimensions. One figure is in the demographic dimension, and the other is in the geographical dimension. As shown in the figures, the Lorenz curves in A are closer to the absolute equality curve. This finding indicates that the mental health beds in the demographic dimension were more equitable than those in the geographical dimension. In terms of time, the Lorenz curves in 2020 were closer to the absolute equality curve than those in 2011. This finding affirms that the equity of mental health beds was better in 2020.

### Gini coefficient

Moreover, Gini coefficients, as shown in Table 2, are used to illustrate the trend of equity in mental health beds from 2011 to 2020. The Gini coefficients by population range between 0.212 and 0.258, which indicates relative equality. However, the Gini coefficients by geographic area range between 0.664 and 0.710, indicating that the distribution of mental health beds exhibits an extreme level of inequity. The Gini coefficients based on both population and geographic area from 2011 to 2020 present a slow downward trend, which indicates that the equality status improved.

**Table 2:** Gini coefficients of mental health bed allocation by population and geographical area (2011–2020)

	2011	2012	2013	2014	2015	2016	2017	2018	2019	2020
Based on population	0.258	0.241	0.225	0.220	0.218	0.226	0.226	0.217	0.218	0.212
Based on area	0.710	0.703	0.698	0.695	0.692	0.690	0.683	0.668	0.665	0.664

### Theil index

Table 3 shows the Theil indices of mental health beds in China from 2011 to 2020. The results are consistent with the results of the Gini coefficient. The Theil index based on area is always higher than that based on population from 2011 to 2020. The decomposition of the Theil index, depicted in Table 3, demonstrates the trends of intraregional and interregional inequity in mental health bed allocation within and among the eastern, central and western regions.

Demographically, in 2020, the Theil index of mental health beds in the eastern region was the smallest, and the largest index was in the western region. This finding means that the allocation of beds in the eastern region is the most equitable, and that in the western region is the least equitable. The Theil index in the eastern region declined from 0.058 to 2011 to 0.014 in 2020, which means that the allocation of beds was improving in the eastern region. T in the central region increased from 0.013 to 2011 to 0.024 in 2020, which means that the allocation of beds worsened in the central region. T in the western region did not change much.

The inequity within these regions were the main cause for the overall inequity. The interregional Theil index for mental health beds declined from 0.008 to 2011 to 0.005 in 2020, and the corresponding intraregional Theil index dropped from 0.042 to 0.028. Despite slightly decreasing trends of both the intraregional and interregional inequity indices of mental health beds, the intraregional Theil indices continued to account for approximately 85 -95% of the total Theil index during the study period. This finding shows that the inequity within these regions were the main cause of the overall inequity. Next, we continue to decompose intraregional differences (Table 4), and we find that the inequity in mental health bed allocation mainly comes from within the eastern region from 2011 to 2015 and within the western region from 2016 to 2020.

Based on area, in 2020, the allocation of beds in the eastern region was the most equitable, while it was the worst in the western region. The Theil index between regions tended to decrease gradually, indicating that interregional inequity narrowed. Moreover, the inequity within regions worsened as the intraregional Theil index increased from 0.538 to 2010 to 0.614 in 2020. As shown in Table 3, the inequity mostly came from intraregional differences as well. The contribution rates of intraregional differences ranged between 69.96% and 81.65%. We found that the mental health bed allocation inequity mostly came from within the western region (Table 4).

**Table 3:** Theil index of mental health bed allocation in the three regions from 2010 to 2020

	Year	East	Central	West	National	Inter-Region (%)	Intra-Region (%
Based on population	2011	0.058	0.013	0.053	0.05	0.008 (16.00)	0.042 (84.00)
	2012	0.048	0.014	0.053	0.044	0.005 (11.36)	0.039 (88.64)
	2013	0.043	0.014	0.052	0.039	0.003 (7.69)	0.036 (92.31)
	2014	0.038	0.019	0.05	0.037	0.002 (5.41)	0.035 (94.59)
	2015	0.035	0.022	0.053	0.037	0.002 (5.41)	0.035 (94.59)
	2016	0.034	0.024	0.056	0.038	0.002 (5.26)	0.036 (94.74)
	2017	0.027	0.026	0.063	0.039	0.003 (7.69)	0.036 (92.31)
	2018	0.021	0.023	0.054	0.035	0.004 (11.43)	0.031 (88.57)
	2019	0.019	0.023	0.058	0.035	0.004 (11.43)	0.031 (88.57)
	2020	0.014	0.024	0.054	0.033	0.005 (15.15)	0.028 (84.85)
Based on area	2011	0.137	0.043	0.72	0.769	0.231 (30.04)	0.538 (69.96)
	2012	0.122	0.048	0.74	0.772	0.221 (28.63)	0.551 (71.37)
	2013	0.113	0.056	0.766	0.782	0.211 (26.98)	0.571 (73.02)
	2014	0.104	0.062	0.788	0.794	0.208 (26.20)	0.586 (73.80)
	2015	0.096	0.068	0.824	0.805	0.193 (23.98)	0.612 (76.02)
	2016	0.09	0.072	0.798	0.774	0.181 (23.39)	0.593 (76.61)
	2017	0.078	0.069	0.838	0.781	0.161 (20.61)	0.62 (79.39)
	2018	0.063	0.075	0.809	0.746	0.147 (19.71)	0.599 (80.29)
	2019	0.059	0.073	0.839	0.76	0.14 (18.42)	0.62 (81.58)
	2020	0.051	0.083	0.83	0.752	0.138 (18.35)	0.614 (81.65)

**Table 4:** Proportion of differences in contribution within the eastern, central and western regions

	Based on population			Based on area		
	Eastern region	Central region	Western region	Eastern region	Central region	Western region
2011	56.48	9.89	33.63	2.82	1.39	95.79
2012	51.55	11.13	37.32	2.47	1.52	96.02
2013	49.15	12.06	38.78	2.20	1.70	96.10
2014	45.18	16.53	38.29	1.96	1.85	96.19
2015	40.68	19.18	40.14	1.74	1.92	96.34
2016	38.36	20.42	41.22	1.68	2.10	96.22
2017	30.87	22.21	46.91	1.39	1.94	96.67
2018	28.29	23.67	48.04	1.16	2.17	96.67
2019	25.21	23.22	51.58	1.06	2.05	96.89
2020	22.02	25.67	52.31	0.92	2.36	96.72

## Discussion

The Chinese government attaches great importance to health care. In recent years, a series of measures have been taken to promote the development of the mental health system. This study shows that the number of mental health bed resources in China increased steadily from 2011 to 2020, and the density of beds increased as well. The implementation of a series of medical reform measures, particularly the Mental Health Prevention and Control System Construction and Development Plan, which was launched by the National Development and Reform Commission, the Ministry of Health, and the Ministry of Civil Affairs in 2010[33], is closely linked to this result. From 2011 to 2015, the central and local governments spent 16.9 billion yuan rebuilding and expanding 549 mental health institutions, equipping 648 mental health institutions with basic medical equipment, and helping general hospitals establish psychiatric departments[34]. Our study discovered that the increase in bed density in the western region is particularly noticeable, reflecting the government’s priority in allocating mental health resources to this region. In fact, the Chinese government enacted the Construction Plan of the National Health Protection Project[35] in 2015 to support the construction of six major projects, including health poverty alleviation, public health service capacity enhancement, and difficult and complicated diseases enhancement, as well as promoting the flow of medical resources to the grassroots level and western regions. This plan requires that new beds be tilted towards areas such as psychiatry when strengthening the construction of county-level hospitals.

The policy initiatives mentioned above have shown good results. However, there remains a disparity in the allocation of mental health beds between China and high-income countries. According to the data of the World Mental Health Atlas 2020, the allocation level of mental health beds in high-income countries is 5.1 per 10,000 people[17], while China has only 4.6 per 10,000 people. In fact, it is inappropriate to compare China to high-income nations due to their disparate economic, cultural and medical systems. And many high-income countries are reducing the number of mental health beds as part of a broader effort to deinstitutionalise mental health patients. However, like many low- and middle-income countries, mental health services in China have a long way to go in achieving the goal of providing good mental health care in the community[5]. With a population of more than 1.4 billion, China has a large demand for mental health bed resources. Especially in the case of the outbreak of the COVID-19 pandemic, sufficient mental health resources become even more crucial[36, 37]. As a result, it is suggested that the government continue to increase the allocation of mental health beds(especially in primary health care) by increasing policy support and financial guarantees. In addition, the impact of the COVID-19 outbreak on the demand for mental health services must be considered.

In this study, the Gini index was used to reflect the overall equality, while the Theil index was used to decompose the sources of inequity. To gain more insight, we evaluated the equality of mental health beds based on both population and geographic area. We found that equity in resource allocation has been improving annually over the past decade. According to population allocation, overall equality is relatively fair. Nonetheless, it is in a highly inequitable state according to geographic area. This result is not surprising since most resource planning programs were based on population allocation, with few focusing on geographic areas. Correspondingly, the equity of resource allocation by population was much better than that by geographical area. Many scholars have reached the same conclusion[7, 30]. However, it is pleasing to note that the “Healthy China 2030” plan, released by the Communist Party of China Central Committee and the State Council, takes fairness and justice as one of the plan’s basic principles[38]. According to the plan, primary health care resources should be distributed fairly based on resident population and service area. As a result, inequity in mental health bed resources by area may improve in the future.

From the perspective of inequity decomposition, mental health bed resources in China are unevenly distributed across regions, and intraregional inequity is the main contributor. The findings are consistent with those of previous studies on the allocation of healthcare resources in terms of expenditure[4], facilities[39, 40], medical equipment[41] and professionals[30]. Moreover, we found that the inequity in mental health bed allocation mostly came from within the western region. By taking the number of mental health beds per 10,000 square kilometres in 2020 as an example, the value of Chongqing was 2400 times that of Tibet. Therefore, the allocation of mental health beds in the western region needs to be optimized. However, this is not an easy job. On the one hand, the western region is vast, but many areas are sparsely populated; on the other hand, the western region is home to ethnic minorities, such as Tibetans and Uyghurs, and has unique cultural-religious characteristics[42]. How mental health services are sought may differ between the western region and the central and eastern regions. For example, residents in the western region may seek help from Tibetan Buddhist or Tibetan traditional medicine when they suffer from illness[43, 44]. Therefore, policymakers should consider all of these factors to promote the rational allocation of mental health services in the western region.

The Chinese government has taken many initiatives and achieved great results in addressing the huge burden of mental disorders. However, China’s mental health service system development and service delivery still face many difficulties[45]. In addition to optimizing mental health beds and associated human resources, timely assessment of needs, appropriate public health policies, development of new psychotropic drugs and effective interventions, strengthening of human capacity and efficient mobilization of financial resources are also important and need to be concerned. Furthermore, information technology positively impacts access to medical care for patients in rural groups and those far from medical resources[46, 47]. Health information interventions, such as telemedicine, internet-based helplines and mental health mobile apps, are expected to achieve health equity and reduce the burden on existing mental health services. In general, promoting health equity is a systemic project. Therefore, it is recommended that the government should follow a holistic approach and consider the whole picture when allocating mental health resources.

## Limitations

It is important to recognize the limitations of this study. First, the research object of this article is the mental health beds in 31 provinces in China. We did not further subdivide mental health beds into different categories. Future studies need to be conducted by focusing on specialized mental health bed categories, such as public and private, profit and nonprofit, mental hospitals, mental health beds in general hospitals and community residential facilities. Second, this study mainly analyses the equity of mental health bed resource allocation from the perspective of demography and geography, without considering the actual mental health status and mental health service needs of different regions. Future studies should consider more factors to comprehensively evaluate mental health bed allocation. Third, in addition to mental health beds, health service indicators, especially the number of health professionals and the amount of public expenditure, should be emphasized in the allocation plan of mental health beds.

## Conclusion

This study’s results show that the total number of mental health beds in China has increased steadily over the past 10 years. However, large gaps still exist in the distribution of mental health bed resources between different regions. The equity of mental health bed resources by population is better than that by geographic area, and the disproportionate distribution of the mental health bed resources within different regions was the main source of inequity. Policymakers need to consider the geographical accessibility of health resources to ensure that people have access to available mental health services. The equality of the allocation of mental health beds within different regions, especially the western region, needs to be optimized.

## Data Availability

Data was collected from China Statistical Yearbook(http://www.nhc.gov.cn/mohwsbwstjxxzx/tjzxtjcbw/tjsj_list.shtml)and China National administrative division information platform(http://xzqh.mcm.gov.cn/map).
